# Association between depression and glycemic control among type 2 diabetes patients in Lima, Peru

**DOI:** 10.1111/appy.12190

**Published:** 2015-06-02

**Authors:** Brenda Crispín‐Trebejo, María Cristina Robles‐Cuadros, Antonio Bernabé‐Ortiz

**Affiliations:** ^1^ Escuela de MedicinaUniversidad Peruana de Ciencias AplicadasLimaPerú; ^2^ CRONICAS Center of Excellence in Chronic DiseasesUniversidad Peruana Cayetano HerediaLimaPerú

**Keywords:** depression, glycated hemoglobin, Peru, prevalence, type 2 diabetes

## Abstract

**Introduction:**

There is limited and controversial information regarding the potential impact of depression on glycemic control. This study aims to evaluate the association between depression and poor glycemic control. In addition, the prevalence of depression and rates of poor glycemic control were determined.

**Methods:**

Cross‐sectional study performed in the endocrinology unit of two hospitals of ESSALUD in Peru. The outcome of interest was poor glycemic control, evaluated by glycated hemoglobin (HbA1c: < 7% versus ≥ 7%), whereas the exposure of interest was depression defined as 15 or more points in the Patient Health Questionnaire‐9 tool.

The association of interest was evaluated using Poisson regression models with robust standard errors reporting prevalence ratios (PR) and 95% confidence intervals (95% CI) adjusting for potential confounders.

**Results:**

A total of 277 participants, 184 (66.4%) males, mean age 59.0 (SD: 4.8), and 7.1 (SD: 6.8) years of disease were analyzed. Only 31 participants (11.2%; 95% CI: 7.5%–14.9%) had moderately severe or severe depression, whereas 70 (25.3%; 95% CI 20.3%–30.8%) had good glycemic control. Depression increased the probability of having poor glycemic control (PR = 1.32; 95% CI 1.15–1.51) after adjusting for several potential confounders.

**Conclusions:**

There is an association between depression and poor glycemic control among type 2 diabetes patients. Our results suggest that early detection of depression might be important to facilitate appropriate glycemic control and avoid further metabolic complications.

## Introduction

Worldwide, there are an estimated 382 million people living with type 2 diabetes (T2D), mostly aged between 40 and 59 years, and causing 5.1 million deaths and at least US$ 548 billion in health care expenditures (International Diabetes Federation, 2013). Moreover, 80% of T2D cases live in low and middle‐income countries (LMIC).

Adequate glycemic control reduces the risk of developing complications such as stroke, cardiovascular events, amputation, and chronic kidney disease (World Health Organization, 2014). Although poor glycemic control has been associated with greater diabetes‐related health care costs (Degli Esposti *et al*., 2013), several studies in different settings have reported suboptimal glycemic control rates among T2D patients (Hu *et al*., 2008; McDonald *et al*., 2009; Li *et al*., 2013). Moreover, a decrease in adequate glycemic control rates has been also observed during past years in some locations (Koro *et al*., 2004).

Diverse factors have been associated with poor glycemic control rates, including younger age (Benoit *et al*., 2005; Rogvi *et al*., 2012), lower education (Rogvi *et al*., 2012), longer duration of diabetes (Benoit *et al*., 2005; Khattab *et al*., 2010), not having health insurance (Benoit *et al*., 2005), but also, emotional distress (Fisher *et al*., 2010; Rogvi *et al*., 2012). Regarding mental health, the presence of depressive symptoms has been also proposed as a potential factor associated with poor glycemic control (Lustman *et al*., 2000; Fisher *et al*., 2010; Heckbert *et al*., 2010). Although a bidirectional relationship of depression and diabetes has been proposed (Golden *et al*., 2008), the prevalence of depression among patients with T2D is relatively high when compared with the general population (Anderson *et al*., 2001; Lustman and Clouse, 2005; Zhang *et al*., 2014), but there is limited and controversial information regarding the potential impact of depression on glycemic control.

Therefore, the aim of this study was to evaluate the association between depression and poor glycemic control after controlling for several potential confounders. In addition, we also determined the prevalence of depression as well as rates of glycemic control among T2D patients.

## Methods

### Study design and settings

Cross‐sectional study performed in the endocrinology unit of two hospitals of the Social Security (ESSALUD) in Peru: Hospital Guillermo Almenara Irigoyen (HGAI, Lima) and Hospital Alberto Sabogal Sologuren (HASS, Callao) between February and September 2014. Both are third‐level hospitals with specialized units and services. HGAI attends 80,000 patients yearly, whereas HASS has 78,000 patients annually (Seguro Social de Salud del Peru, 2014).

### Study population and selection criteria

Patients with diagnosis of type 2 diabetes mellitus, aged between 30 and 65 years old, with recent results of glycated hemoglobin (HbA1c), were invited to participate. Illiterate patients and non‐Spanish speakers were excluded from the study.

### Variables definition

The outcome of interest was poor glycemic control, assessed using HbA1c. Data were collected from clinical records up to three months before the date of interview as HbA1c reflects average plasma glucose over that previous time in a single measure (World Health Organization, 2006). A cutoff of 7% (53 mmol/mol) was used to define good and poor glycemic control (<7% versus ≥ 7%) based on international classification (Expert Committee on the Diagnosis and Classification of Diabetes Mellitus, 2003).

The exposure of interest was depression. The Patient Health Questionnaire (PHQ‐9), developed by Kroenke *et al*. (2001) and validated in Spanish (Baader *et al*., 2012) was used in this study. The sensitivity and specificity of this tool has shown to be as high as 92% and 89%, respectively, in detecting depressive patients (Baader *et al*., 2012). The instrument comprised nine questions, each one with four options of response: not at all, several days, more than half the days, and nearly every day with scores between zero and three points (total score between zero and 27 points). A cutoff of 15 or more was used to establish clinical depression, as this score warrants treatment for patients (antidepressants, psychotherapy, or a combination of therapies as reported elsewhere) (Kroenke *et al*., 2001).

Sociodemographic variables included gender (male or female), age (<60 or ≥60 years), education level (primary, secondary, or superior), work status (no or yes), place of birth (Lima/Callao or other places), and hospital settings (HGAI or HASS). In addition, clinical history variables were evaluated such as time of disease, in years, and categorized in <5, 5–9, or ≥10 years; hospital status, evaluated as outpatients or inpatients; last year number of hospital admissions, categorized in 0, 1, 2, or ≥3; as well as hypertension diagnosis (no or yes), retinopathy diagnosis (no or yes), and diabetic foot diagnosis (no or yes). Finally, laboratory results were also assessed including total cholesterol, defined as <200 or ≥200 mg/dL, according to the Adult Treatment Panel III (National Cholesterol Education Program [NCEP] Expert Panel on Detection; Evaluation; and Treatment of High Blood Cholesterol in Adults (Adult Treatment Panel III, 2002), and 24‐hour protein levels in urine, split in <0.5, 0.50–0.99, 1.00–1.99, and ≥2 grams in 24 hours (Ivanyi *et al*., 2001).

### Procedures and sample collection

Potential participants were contacted in the endocrinology unit of the involved hospitals during their clinical routine appointments (outpatients) or during current hospitalization (inpatients). Patients were recruited using a non‐probabilistic technique, consecutively as they presented in the consulting room or in the hospitalization area. After oral informed consent, a face‐to‐face questionnaire was applied containing detailed information regarding sociodemographics, clinical history, and information regarding depressive symptoms. The most recent laboratory results, including glycated hemoglobin (HbA1c), total cholesterol levels, and 24‐hour protein in urine values, were obtained from clinical records.

### Sample size

Sample size was calculated using the statistical package Power and Sample Size (PASS 2008, NCSS, Utah, USA). Using a 5% significance level and 80% of power, 272 participants were required to find a strength of association ≥ 2.5, assuming that 73.6% of patients has poor glycemic control (HbA1c <7%) (Lazo Mde *et al*., 2014), and 16.9% had depression (Balhara and Sagar, 2011).

### Statistical analysis

After data collection, a double data entry process was performed using Microsoft Excel for Windows. Then, data were transferred to STATA 13 (STATA Corp, College Station, TX, USA) for statistical analysis. First, a description of the study population was performed using proportions to compare population characteristics according to the presence of depression and glycemic control, our variables of interest. Comparisons between variables were performed using Chi‐squared test of Fisher's exact test accordingly. Second, prevalence and 95% confidence intervals (95% CI) of the variables of interest were calculated. Given the cross‐sectional nature of the study, as well as potential high prevalence of the outcome of interest (Barros and Hirakata, 2003), the association between depression and glycemic control was evaluated using Poisson regression models with robust standard errors (Coutinho *et al*., 2008), reporting prevalence ratios (PR) and 95% CI adjusted for potential confounders. Different models were fitted utilizing a hierarchical approach (Victora *et al*., 1997), conducted to better understand the potential influence of covariates in the association of interest.

### Ethics

This project was reviewed and approved by the Ethical Committees of the Universidad Peruana de Ciencias Aplicadas, Lima, Peru. In addition, protocol and informed consents were approved by Ethical Boards in each of the participant hospitals. An oral informed consent was utilized to explain the purpose of the study. Data were collected without personal identifiers to guarantee appropriate confidentiality.

## Results

### Characteristics of the study population

A total of 320 participants were contacted, but only 305 (rejection rate: 4.7%) accepted participation and were then evaluated. Of them, 28 (9.2%) were excluded from further analyses because of incomplete information or data with inconsistencies. Sociodemographic characteristics of those included and excluded from analysis were not different (data not shown). Thus, 277 participants, 184 (66.4%) males, mean age 59.0 (SD: 4.8), and 7.1 (SD: 6.8) years of disease were analyzed.

### Prevalence of depression and associated factors

Based on PHQ‐9 results, 31 participants (11.2%; 95% CI 7.5%–14.9%) presented moderately severe or severe depression. Only being currently working (*P* = 0.04), place of birth (*P* = 0.02), and hospital setting (*P* = 0.01) were associated with depression. Detailed information of the population characteristics according to the presence of depression is shown in Table 1.

**Table 1:** Characteristics of the study population according to depressive symptoms

	Depression		*P*‐value*
	No (*n* = 246)	Yes (*n* = 31)	
Gender			
Males	165 (67.1%)	19 (61.3%)	0.52
Females	81 (32.9%)	12 (38.7%)	
Age			
<60 years	120 (48.8%)	11 (35.5%)	0.16
≥60 years	126 (51.2%)	20 (64.5%)	
Education level			
Primary	50 (20.3%)	5 (16.2%)	0.72
Secondary	86 (35.0%)	13 (41.9%)	
Superior	110 (44.7%)	13 (41.9%)	
Work status			
No, currently working	61 (24.8%)	13 (41.9%)	0.04
Yes, currently working	185 (75.2%)	18 (58.1%)	
Place of birth			
Lima/Callao	141 (57.3%)	11 (35.5%)	0.02
Other places	105 (42.7%)	20 (64.5%)	
Hospital setting			
HGAI	159 (64.6%)	13 (41.9%)	0.01
HASS	87 (35.4%)	18 (58.1%)	
Time of disease			
<5 years	104 (42.3%)	11 (35.5%)	0.75
Between 5 and 9 years	82 (33.3%)	11 (35.5%)	
≥10 years	60 (24.4%)	9 (29.0%)	
Hospital status			
Outpatient	213 (86.6%)	27 (87.1%)	0.99
Inpatient	33 (13.4%)	4 (12.9%)	
Last year, hospital admissions			
0	43 (17.5%)	5 (16.1%)	0.87
1	114 (46.3%)	14 (45.2%)	
2	65 (26.4%)	10 (32.3%)	
≥3	24 (9.8%)	2 (6.4%)	
Hypertension diagnosis			
No	104 (42.3%)	11 (35.5%)	0.47
Yes	142 (52.7%)	20 (64.5%)	
Retinopathy			
No	218 (88.6%)	25 (80.7%)	0.24
Yes	28 (11.4%)	6 (19.4%)	
Diabetic foot			
No	152 (61.8%)	16 (51.6%)	0.27
Yes	94 (38.2%)	15 (48.4%)	
Total cholesterol			
<200 mg/dL	167 (67.9%)	17 (54.8%)	0.15
≥200 mg/dL	79 (32.1%)	14 (45.2%)	
24‐hour proteins in urine			
<0.5 grams in 24 hours	80 (32.5%)	10 (32.3%)	0.68
0.50 to 0.99 grams in 24 hours	119 (48.4%)	15 (48.4%)	
1.00 to 1.99 grams in 24 hours	28 (11.4%)	2 (6.5%)	
≥2 grams in 24 hours	19 (7.7%)	4 (12.9%)	

*Comparisons were performed using Chi‐squared test, except in the case of hospital status and retinopathy, where Fisher's exact test was used instead.

### Glycemic control and associated factors

Seventy participants (25.3%; 95% CI 20.3–30.8%) had good glycemic control based upon levels of glycated hemoglobin. In bivariable model, factors associated with adequate glycemic control were: age (*P* = 0.03), hospital setting (*P* < 0.001), time of disease (*P* = 0.002), number of hospital admissions in last year (*P* < 0.001), retinopathy diagnosis (*P* = 0.02), total cholesterol (*P* = 0.01), and 24‐hour proteins in urine (*P* < 0.001). Information is presented in Table 2.

**Table 2:** Association between glycemic control and population characteristics

	Glycemic control		*P*‐value*
	<7% (*n* = 70)	≥ 7% (*n* = 207)	
Gender			
Males	42 (60.0%)	142 (68.6%)	0.19
Females	28 (40.0%)	65 (31.4%)	
Age			
<60 years	41 (58.6%)	90 (43.5%)	0.03
≥60 years	29 (41.4%)	20 (56.5%)	
Education level			
Primary	8 (11.4%)	47 (22.7%)	0.11
Secondary	26 (37.1%)	73 (35.3%)	
Superior	36 (51.4%)	87 (42.0%)	
Work status			
No currently working	23 (32.9%)	51 (24.6%)	0.18
Yes, currently working	47 (67.1%)	156 (75.4%)	
Place of birth			
Lima/Callao	43 (61.4%)	109 (52.7%)	0.20
Other places	27 (38.6%)	98 (47.3%)	
Hospital setting			
HGAI	31 (44.3%)	141 (68.1%)	<0.001
HASS	39 (55.7%)	66 (31.9%)	
Time of disease			
<5 years	41 (58.6%)	74 (35.7%)	0.002
Between 5 and 9 years	14 (20.0%)	79 (38.2%)	
≥10 years	150 (21.4%)	53 (26.1%)	
Hospital status			
Outpatient	62 (88.6%)	178 (86.0%)	0.58
Inpatient	8 (11.4%)	29 (14.0%)	
Last year, hospital admissions			
0	27 (38.6%)	21 (10.1%)	<0.001
1	31 (44.3%)	97 (46.9%)	
2	8 (11.4%)	67 (32.4%)	
≥3	4 (5.7%)	22 (10.6%)	
Hypertension diagnosis			
No	36 (51.4%)	79 (38.2%)	0.05
Yes	34 (48.6%)	128 (61.8%)	
Retinopathy			
No	67 (95.7%)	176 (85.0%)	0.02
Yes	3 (4.3%)	31 (15.0%)	
Diabetic foot			
No	43 (61.4%)	125 (60.4%)	0.88
Yes	27 (38.6%)	82 (39.6%)	
Total cholesterol			
<200 mg/dL	55 (78.6%)	129 (62.3%)	0.01
≥200 mg/dL	15 (21.4%)	78 (37.7%)	
24‐hour proteins in urine			
<0.5 grams in 24 hours	35 (50.0%)	55 (26.6%)	<0.001
0.50 to 0.99 grams in 24 hours	30 (42.9%)	104 (50.2%)	
1.00 to 1.99 grams in 24 hours	1 (1.4%)	29 (14.0%)	
≥2 grams in 24 hours	4 (5.7%)	19 (9.2%)	
Depression			
No	68 (97.1%)	178 (86.0%)	0.01
Yes	2 (2.8%)	29 (14.0%)	

*Comparisons were performed using Chi‐squared test.

HASS, Hospital Alberto Sabogal Sologuren; HGAI, Hospital Guillermo Almenara Irigoyen.

### Depression and glycemic control

Among those with moderately severe or severe depression, only 6.4% participants had good glycemic control, compared to 27.6% among those without depression (*P* = 0.01). Depression increased the probability of having poor glycemic control (PR = 1.32; 95% CI 1.15–1.51) after adjusting for several potential confounders (Table 3).

**Table 3:** Association between depressive symptoms and glycemic control: crude and adjusted models

Depression	Association models				
	Crude model	Model 1	Model 2	Model 3	Model 4
	PR (95% CI)	PR (95% CI)	PR (95% CI)	PR (95% CI)	PR (95% CI)
No	1 (reference)	1 (reference)	1 (reference)	1 (reference)	1 (reference)
Yes	1.29 (1.15–1.46)	1.39 (1.21–1.59)	1.36 (1.20–1.54)	1.34 (1.18–1.53)	1.32 (1.15–1.51)

Model 1: adjusted by gender, age, education level, time of disease, working currently, place of birth, and hospital setting.

Model 2: adjusted by variables in Model 1 plus hospital status, and last year hospital admissions.

Model 3: adjusted by variables in Model 2, plus hypertension diagnosis, retinopathy diagnosis, and diabetic foot diagnosis.

Model 4: adjusted by variables in Model 3, plus total cholesterol and 24‐hour proteins in urine.

CI, confidence interval; PR, prevalence ratio.

## Discussion

### Main findings

This study shows a strong relationship between having depression and poor glycemic control. Thus, patients with depression had about 1.3 times greater prevalence of poor glycemic control that those without depression after controlling for potential confounders. In addition, more than one in 10 T2D cases presented clinical depression, and only about a quarter of patients had good glycemic control.

### Depression and glycemic control: comparison with other studies

There are several cross‐sectional studies reporting that depression is a factor associated with poor glycemic control among patients with T2D (Lustman *et al*., 2000; Lustman and Clouse, 2005; Papelbaum *et al*., 2011; Zhang *et al*., 2014). Our results are consistent with these findings even after controlling for a large list of potential confounders; our hierarchical approach is reassuring, suggesting that the impact of covariates is not considerable in the proposed association as estimates remain roughly constant in the different models. On the other side, there are also cross‐sectional studies reporting that diabetes‐related distress and not depressive symptoms were associated with poor glycemic control among Japanese patients with T2D (Tsujii *et al*., 2012). This latter paper, however, defined poor glycemic control as HbA1c ≥8% instead of traditional 7% and controlled results by body mass index, a marker we could not include in our models due to unavailability. In addition, we decided to use a more conservative definition of depression (cutoff of 15 or more points) due to the fact that PHQ‐9 tool warrants treatment and/or psychotherapy for these patients (clinical depression), avoiding potential misclassification; we also conducted post hoc analyses using different cutoffs with PHQ‐9 score; e.g., 10–14 (moderate depression) and 15 and more points (moderately severe and severe depression) compared to those with 0–9 (normal), and found an increasing trend in the magnitude of association estimates (from a PR = 1.23 among those with 10–14 points to a PR = 1.40 among those with 15 or more points, *P*‐value for trends < 0.001, data not shown). Nevertheless, previous results from longitudinal studies showed that diabetes distress linked specifically to diabetes and its management, but not clinical depression or depressive symptoms seems to be associated with glycemic control (Fisher *et al*., 2010). Additionally, another longitudinal study shows that the adverse effect of depression on outcomes in T2D patients might be not mediated by glycemic, blood pressure or lipid control (Heckbert *et al*., 2010). However, both studies evaluated depression in one point in time or after a longer period (after 18 months), when mood, and for instance depression, can change over time. Therefore, we believe results are still controversial. In addition, the association of diabetes and depression is thought to be bidirectional (Golden *et al*., 2008), and therefore depression can increase the risk of diabetes, and diabetes increases also the risk of developing depression.

### Prevalence of depression and poor glycemic control

Prevalence of depression among T2D patients is high when compared with general population (Huang *et al*., 2006; Heckbert *et al*., 2010; Al‐Amer *et al*., 2011), varying from 5% to 60% according to the results of a previous meta‐analysis (Anderson *et al*., 2001). Our results found that more than one in 10 patients presented moderately severe or severe depression; although when the usual PHQ‐9 cutoff of 10 was used, prevalence increased to 33.6% (data not shown). These results suggest the need for a screening assessment of T2D cases during routine evaluation as patients are periodically contacting the health system. Even so, the mechanisms by which depression and T2D are linked remain unclear.

On the other hand, rates of poor glycemic control are similarly consistent with previous international (Koro *et al*., 2004; Degli Esposti *et al*., 2013) and national (Lerner *et al*., 2013; Lazo Mde *et al*., 2014) reports. Results, however, can vary according to the type of patients assessed. Thus, when patients are included from the general population, rates of poor glycemic control are lower (Silva *et al*., 2010; Lerner *et al*., 2013) when compared to patients recruited from hospitals and health facilities (Lopez Stewart *et al*., 2007; Lazo Mde *et al*., 2014). In any case, glycemic control is low, carrying out particular challenges especially in resource‐constrained settings including rate of complications, number of hospitalizations, as well as out‐of‐pocket payments to cover health care.

### Strength and limitations

The present study benefits from using a validated instrument to assess depression, imposing a cutoff to better detect participants with depression, as well as the large number of confounders including in the models created utilizing a hierarchical approach to disentangle potential impact of these covariates. However, this study has several limitations. First, the study design, cross‐sectional in nature, can only determine association and not causality. Although we used regression models and adjusted for several confounders, longitudinal studies, with repeated participants' measures, are needed to corroborate our findings. Second, the HbA1c value was not obtained at the same time as the mental health assessment; however, we believe that the potential impact of this can be negligible as glycated hemoglobin reflects mean plasma glucose over the previous two to three months (World Health Organization, 2006). In addition, our estimates are in line with previous works. Third, although our models included a large list of confounders, variables such as socioeconomic status or overweight/obesity were not included. However, education has been suggested as a good form to assess socioeconomic status (Howe *et al.,* 2012). Fourth, patients enrolled in the study were drawn from hospital clinical settings instead from the general population; hence, some selection bias might arise, especially in the case of depression rates. Finally, the PHQ 9 is a screening tool for depression; as a result, some risk of misclassification might arise despite of the high sensitivity and specificity of the tool.

In conclusion, there is an association between depression and poor glycemic control among T2D patients. Our results suggest that early detection of depression might be important to facilitate appropriate glycemic control and thus avoid further metabolic complications.

## Conflict of interests

The authors declare they have no conflict of interest.
